# Case report: “an unexpected origin of coma in a young adult”

**DOI:** 10.1186/s12245-021-00390-5

**Published:** 2021-11-27

**Authors:** Ranjana Chandrikasing, Sudeshkoemar Ramnarain, Rakesh Bansie, Harvey Yang, Huibert Ponssen, Navin Ramdhani

**Affiliations:** 1 Intensive Care at Academic Hospital Paramaribo, Paramaribo, Suriname; 2 Resident Emergency Medicine at Academic Hospital Paramaribo, Paramaribo, Suriname; 3 Internal Medicine Department at Academic Hospital Paramaribo, Paramaribo, Suriname; 4 Neurology Department at Academic Hospital Paramaribo, Paramaribo, Suriname; 5 Intensive Care at Albert Schweitzer Hospital Dordrecht, Dordrecht, The Netherlands; 6 Intensive Care Department at Academic Hospital Paramaribo, Paramaribo, Suriname

**Keywords:** Coma, Carbon dioxide narcosis, Non-traumatic quadriplegia, Longitudinally extensive transverse myelitis

## Abstract

We report a peculiar case of acute non-traumatic coma due to neuromuscular hypoventilation syndrome caused by a non-traumatic spinal cord injury (NTSCI). A 21-year-old patient presented to the emergency room complaining of sudden onset weakness in his lower limbs and shortness of breath. While in the ER, he briefly became comatose and labs revealed an acute respiratory acidosis. Detailed neurologic examination ultimately revealed upper motor neuron signs and quadriplegia. He was ultimately diagnosed with a non-traumatic spinal cord injury, in particular, a cervical transverse myelitis which had caused acute diaphragmatic weakness. Although a very rare cause of coma, emergency medicine physicians need to be aware of transverse myelitis, a disorder that may result in rapidly progressive neurologic decline and is treated with immunomodulation.

## Background

Coma is defined as a state of deep unconsciousness, an eyes-closed unresponsive state. Coma is usually a transitory state though it may last for an indefinite or even prolonged period. Alerting and arousal functions of the brain are affected as well as awareness and the content of consciousness. Coma may occur as a complication of an underlying illness, or as a result of injuries [1, 2].

Although there is still some ongoing debate regarding the aforementioned definition, we would like to point out that when we use the word “coma” in our case report, we mainly refer to the initial unresponsiveness of our patient.

Some of the more common causes of coma, likely to be encountered in the Emergency Department are noted in Table 1 [3].

**Table 1:** Differential diagnosis of unresponsiveness [3]

Primary CNS disease or trauma	Causes affecting the brain diffusely
o Direct CNS trauma - Diffuse axonal injury - Subdural hematoma - Epidural hematoma o Vascular disease - Intraparenchymal hemorrhage - Ischemic stroke o CNS infections o Neoplasms o Seizures - Nonconvulsive status epilepticus - Postictal state	o Encephalopathies - Hypoxic encephalopathy - Metabolic encephalopathy - Hypertensive encephalopathy o Hypoglycemia o Hyperosmolar state (e.g., hyperglycemia) o Electrolyte abnormalities (e.g., hypernatremia, hypercalcemia) o Organ system failure - Hepatic encephalopathy - Uremia/renal failure o Endocrine (e.g., Addison’s disease and hypothyroidism) o Hypoxia o Carbon dioxide narcosis o Toxins o Drug reactions (e.g., neuroleptic malignant syndrome) o Environmental causes – hypothermia, hyperthermia o Deficiency state – Wernicke’s encephalopathy o Sepsis

The most common and reversible etiology of coma is metabolic (including drug overdose) followed by structural disorders of the central nervous system [4].

In this particular case report, we describe a peculiar case of non-traumatic coma due to a carbon dioxide narcosis [5], which was caused due to a cervical LETM.

## Case presentation

A 21-year-old African-Surinamese male with no significant medical history presented to the Emergency Room (ER) of the Academic Hospital Paramaribo, with an acute onset of shortness of breath and weakness of the legs within an hour after physical exercise (weight lifting).

Upon arrival in the ER, his vital signs were BP 168/99 mmHg, RR 33/min, HR 100/min, saturation of 99%, temperature of 37.6 °C, and a Glasgow Coma Scale (GCS) of E4M6V4, with a MRC muscle power scale of 4/5 in all extremities.

Shortly after arrival, the patient collapsed and appeared in respiratory distress due to rapid desaturation and a sudden drop in GCS (E1M1V1), while maintaining cardiac output. The patient was intubated using etomidate and succinylcholine.

His initial (venous) blood gas revealed a severe acute respiratory acidosis: pH 6.91, pCO2 167 mmHg, pO2 53 mmHg, sO2 55.2%, base excess 0.5 mmol/L, HC03^-^ 17.7 mmol/L. Glucose and lactate were 9.5 mmol/L and 1.0 mmol/L, respectively.

After ventilation via endotracheal tube over a period of approximately 10 min, the patient regained consciousness and was able to communicate through eye movements. A repeat VBG, showed near normal parameters: pH 7.23, pCO2 59 mmHg, pO2 47.8 mmHg, sO2 66.1%, base excess − 1.8 mmol/L, HC03^-^ 21 mmol/L.

Due to a lack of space on the intensive care unit, as well as the fact that there was only one ventilator on the ER at that time, the decision was made to extubate the patient seeing how he had regained consciousness and his venous blood gas normalized.

However, within seconds, the patient started desaturating, with no visible chest excursions. Once more, a rapid sequence intubation was performed, keeping the patient on the ventilator pending definite transfer to the ICU.

A complete blood cell count, basic metabolic profile, and urinalysis were performed; the only abnormalities noted were an elevated AST 293 IU/L (0–38), ALT 312 IU/L (0– 41), LDH 372 IU/L (98–192), and serum CPK 22345 IU/L (38–174 IU/L), and the latter was attributed to his bodybuilding exercise.

Both an ECG and echocardiography were normal. A computed tomography (CT) of the head only noted a sinusitis, while a CT of the spine, chest, and abdomen showed no mass lesions or other anomalies.

In-depth neurologic examination on the ICU revealed the following:

The patient was intubated, but conscious and aware of his surroundings. He was able to communicate with us using head and eye gestures. Cranial nerve functions 2–12 were intact and both pupils were equal and reactive to light. The patient appeared to be quadriplegic. The tonus of his left leg (MRC grade ^1^/5) which was present upon admission disappeared after 1 day. Patellar reflexes were present, while plantar reflexes were absent.

Further physical examination was normal. There were no palpable masses in the neck, axilla, inguinal regions, and testicles.

Quadriplegia without a cause led to further investigation; spinal cord infections, Guillain–Barré syndrome (GBS), spinal cord hemorrhage or thrombosis of the anterior spinal artery, CNS tumor, and myelitis were our leading possibilities.

A lumbar puncture (opening pressure 3.5 cm, glucose 4.5 mmol/l [2.2–3.9], protein 0.50 g/l [0.15–0.40], cells 3× 10^6/l, polynuclear 100%), was inconclusive for infectious myelitis. Other infectious etiologies for infectious myelitis and GBS (such as ZIKA, HIV, syphilis, HSV I–II, VZV, mycoplasma, SARI) were excluded.

**Fig. 1:**
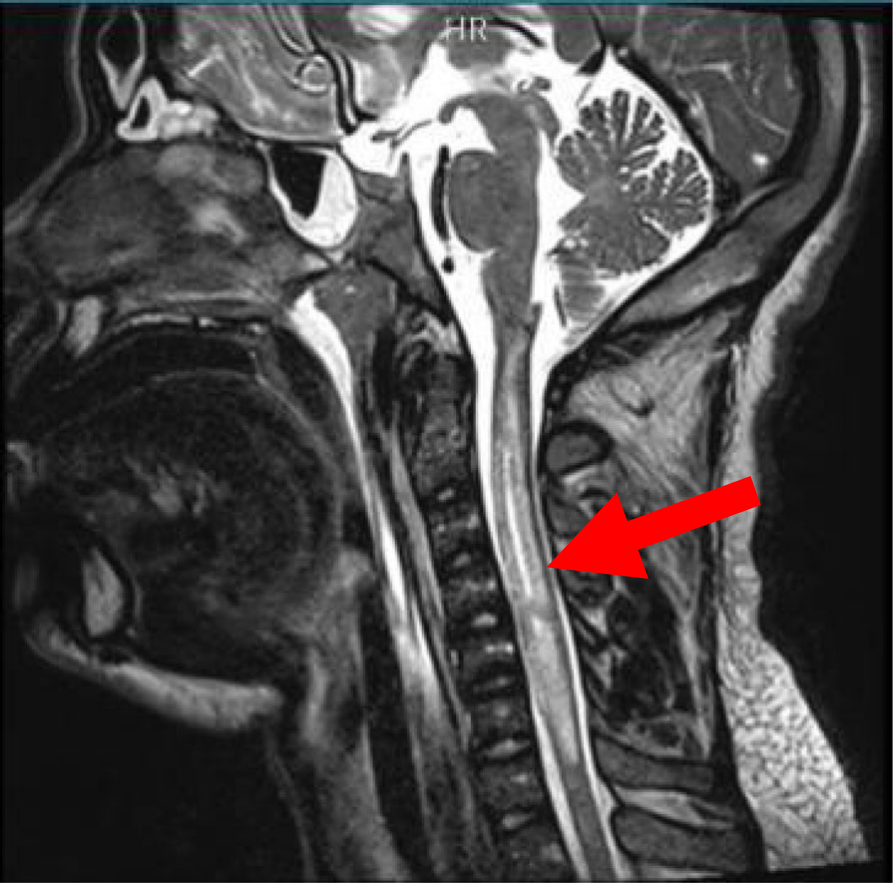
T2_tse sagittal images of the cervical spine showing major edema and minimal syrinx formation of the myelum extending all the way from C1 to C6

A gadolinium-enhanced magnetic resonance imaging (MRI) of the cervical spine showed major edema of the myelum with minimal syrinx formation extending from C1 all the way to C6, suggestive for longitudinal extensive transverse myelitis (LETM) (Fig. 1). No obvious tumors could be seen; however, there was a notable extensive pansinusitis.

Considering the foresight for the necessity of long-term mechanical ventilation, the patient received a tracheostomy.

Given the finding of LETM on the MRI, serological evaluation of auto-immune markers^1^ was pursued, which only noted a weak positive anti-ß2 glycoprotein and an indifferent lupus. Other markers, such as ANF/ENA-6/anticardiolipin, c-ANCA and p-ANCA, anti-AQP4, and anti-MOG, were negative.

Considering LETM as a possible diagnosis, due to an auto-immune disorder, immunosuppressive therapy with cyclophosphamide was initiated alongside corticosteroids followed by intravenous immunoglobulin (IVIg). Due to a pancytopenia, following cyclophosphamide administration, this was suspended after only one dose.

The ophthalmologist found no abnormalities to the nervus opticus or otherwise (such as optic disc swelling, optic neuritis, uveitis, decreased visual acuity and/or field, and iris roseolae)

Some improvement of his motor function in his left leg was seen nine days after receiving his first course of IVIg, while still requiring ventilator support.

A second dose of methylprednisolone was given for 5 days of 1000 mg daily. The patient began to show some spontaneous breath on the ventilator on the last day of his second dose of IVIg.

**Fig. 2:**
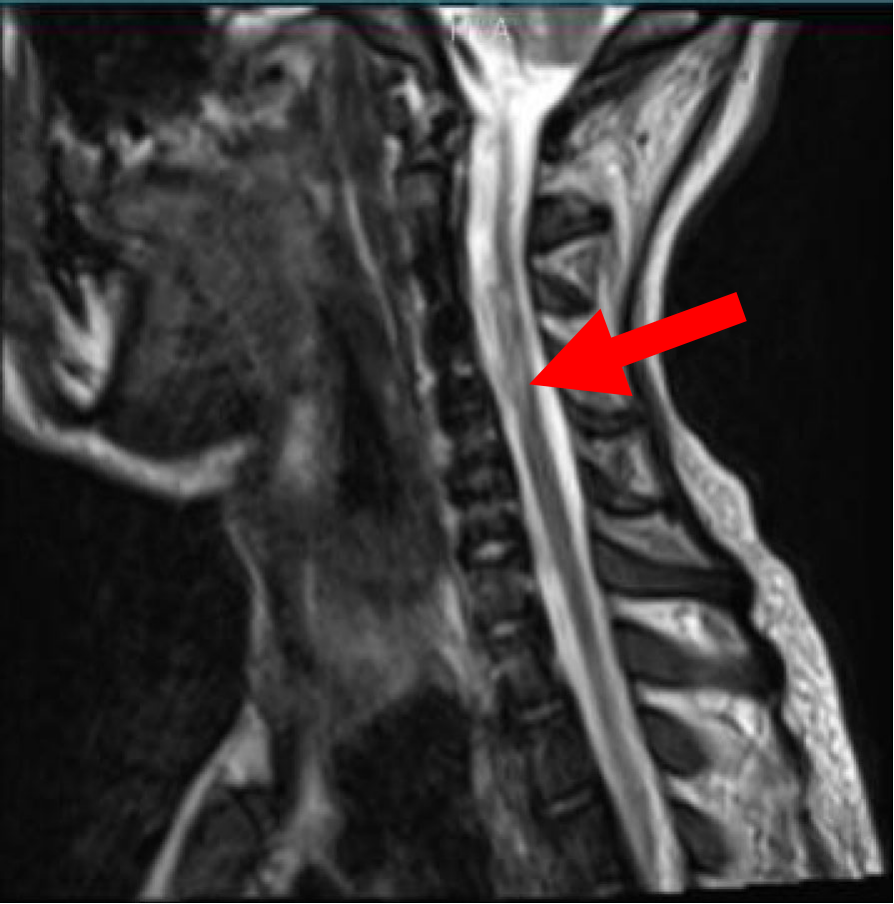
Repeat MRI T2_tse sagittal images, 1 month after starting IVIG and corticosteroid therapy

A repeat MRI (Fig. 2) was done one month after receiving treatment immunoglobulins and high dose corticosteroids, which showed complete resolution of edema, however, with signs of residual myelum damage.

Slowly the patient started showing signs of motor improvement, starting on the left side. Gradually the right side has been showing some improvement, and the patient has been able to sit up straight in bed.

After approximately 5 and a half months, weaning protocol for ventilator support could finally be initiated. He was able to successfully wean over a period of 2 weeks; however, this success was cut short due to multiple ICU-related complications as well as a tracheal laceration causing air leakage and depression.

## Outcome

The patient responded moderately to the combination of IVIg and IV corticosteroids, and poorly to physical therapy. He was ultimately ventilator-dependent in our hospital for many months and continues to have a poor prognosis.

Due to the absence of specialized nursing homes, within the country, this patient still remains admitted to the ICU; treatment was continued with immunosuppressive agents (azathioprine) alongside intensive physiotherapy and psychological guidance.

## Discussion and conclusion

We present a very uncommon clinical manifestation of a non-traumatic spinal cord injury (NTSCI), which initially started with a generalized weakness of the extremities and rapidly progressed to a comatose state as a result of a carbon dioxide narcosis. During the course of the illness of our patient, the NTSCI resulted to be the product of a cervical transverse myelitis (specifically LETM).

Initial management in the Emergency Department (ED) for patients suspected of NTSCI depends on the cause. Usual investigations are imaging (including magnetic resonance) and blood tests and, sometimes, more invasive procedures such as lumbar punctures or angiograms [6].

Causes of NTSCI are listed in Table 2.

**Table 2:** Causes of NTSCI [6]

Etiology or cause	Example
Spinal tumor	Primary tumor (intradural or extradural) Lymphoma Metastases from lung, breast, bowel, or prostate cancer
Degenerative disorders	Degenerative disc disease Herniated disc Spinal stenosis
Vascular	Spinal stroke Aneurysm Hematoma
Inflammatory/autoimmune	Transverse myelitis

Our patient was ultimately diagnosed with a specific form of transverse myelitis, known as longitudinally extensive spinal cord lesions (LESCL) or LETM. It’s characterized by extensive involvement of the spinal cord, with abnormal T2 signal traversing at least three vertebral body segments in length [7, 8].

Epidemiology suggests an incidence of one to eight people per million, with a bimodal peak between the ages 10 to 19 years and 30 to 39 years, and no gender or familial predisposition. However, women did predominate among the cases of TM, mostly associated with multiple sclerosis [8].

### Differential diagnosis [7]

LETM is typical for neuromyelitis optica (NMO) but can be seen in a number of other conditions, as listed in Table 3:

**Table 3:** Differential diagnosis for transverse myelitis [7, 9]

Demyelinating disorders	• Neuromyelitis optica (NMO) o Optico-spinal form of Asian MS (OSMS) • Multiple sclerosis o Confluent short segment lesions mimicking LESCL • Anti-MOG associated encephalomyelitis • Autoimmune GFAP astrocytopathy • Acute disseminated encephalomyelitis (ADEM)
Inflammatory/autoimmune/dysimmune	• Neurosarcoidosis • Sjögren syndrome • Systemic lupus erythematosus (SLE) • Behçet disease • Mixed connective tissue disease (MCTD) • Antiphospholipid antibody syndrome
Vascular	• Spinal cord infarction • Dural arteriovenous fistula (dAVF)
Post-infectious	• Bacterial o *Treponema pallidum, Mycobacterium tuberculosis, Borrelia burgdorferi, Camylobacter jejuni, Acinetobacter baumanii,Leptospira, Chlamydia pneumoniae, Legionella pneumonia, Salmonella paratyphi B, Brucellosis melitensis, Groups A and B streptococci.* • Viral o *Herpes simplex virus type-2 (HSV), Varicella-zoster virus (VZV), Cytomegalovirus (CMV), Dengue virus, Epstein-Barr virus (EBV), Hepatitis A and C, Measles, Mumps, Influenza A virus (including H1N1), Coxasckieviruses A and B, Enterovirus-70 and -71, Echoviruses, Poliovirus 1, 2 and 3, West Nile virus, Japanese encephalitis virus, Tick-borne encephalitis virus, St. Louis encephalitis virus* • Parasitical o *Neurocystercosis, Schistosoma, Echinococcus granulosus, Toxoplasma gondii, Acanthamoeba species, Paragonimus westermani, Trypanosoma brucei, Gnasthostoma angio-strongylosis* • Fungal o *Actinomyces, Coccidioides immitis, Aspergillus, Blastomyces, Cryptococcus*
Other causes	• Paraneoplastic myelitis • Idiopathic (aquaporin-4 negative)

Over the past decade, only a few cases have been reported concerning transverse myelitis, the majority of which involved the thoracic-lumbar region [10–19].

The pathogenesis of transverse myelitis is unknown, although it is noted to follow viral infection in approximately 30% of patients and commonly is termed post-infectious myelitis. Other postulated etiologic categories include infectious, autoimmune, and idiopathic. It can also be seen with a wide variety of connective tissue diseases, such as lupus, Sjögren’s syndrome, antiphospholipid syndrome, and other mixed connective tissue diseases. No apparent cause of acute transverse myelitis is identified in 30% of the patients. Progression of symptoms usually is rapid, with 66% of the cases reaching maximal deficit by 24 h. Symptoms may progress, however, over days to weeks. The thoracic cord region is affected in 60% to 70% of cases. The cervical spinal cord is rarely affected [20].

The first step in the treatment of TM is to exclude treatable causes such as infections and tumors. Based on the underlying pathology, the appropriate treatment should be initiated (e.g., surgery for a compressive lesion, antibiotics for infections, and immunosuppressant for collagen vascular diseases) [21].

Once the secondary causes of TM have been excluded, patients should be treated with intravenous corticosteroids (methylprednisolone 1 g intravenously daily for 5 days. Treatment with intravenous corticosteroids is usually associated with significant improvement of the clinical manifestations but has no significant impact on the long-term prognosis [21].

Prognosis of TM is variable; usually one-third of all patients completely recover, one-third have only partial recovery, and one-third will not improve. Patients with partial transverse myelitis have a more favorable prognosis than those with complete transverse myelitis [21].

Poor prognostic factors include a sudden catastrophic onset, profound weakness, sensory abnormalities at cervical levels, presence of spinal shock, incontinence, and no improvement after 3 months [21].

The clinical course of acute transverse myelitis varies widely, ranging from complete recovery to death from progressive neurologic compromise. Most patients with idiopathic disease have at least partial recovery, which usually begins within 1 to 3 months. Maximal improvement usually is obtained within 3 to 6 months with 30% of patients having a good recovery, 25% a fair recovery, and 30% a poor outcome; there is 15% mortality at 5 years [20].

An unfortunate event was the fact that the decision was made to extubate the patient without having initially found the cause for his carbon dioxide narcosis. Since the patient had reached the ED standing on his own without any support, no in debt neurological exam was performed post intubation, once he regained consciousness.

We conclude that acute hypercapnic coma and quadriplegia in young, previously healthy, patients should prompt ED physicians to consider being in the presence of a non-traumatic spinal cord injury after having excluded the more common causes of coma.

## Footnotes

1 Due to the limited resources available, anti-AQP4, anti-MOG are sent abroad and results take up to 2 weeks before being available.
